# Aging in Down Syndrome: Latest Clinical Advances and Prospects

**DOI:** 10.3390/jcm10215037

**Published:** 2021-10-28

**Authors:** Alessandra C. Martini, Melissa J. Alldred, Ann-Charlotte Granholm

**Affiliations:** 1 Department of Pathology and Lab. Medicine, University of California Irvine, Irvine, CA 92697, USA; ac.martini@uci.edu; 2 Nathan Kline Institute, NYU Grossman Medical School, 140 Old Orangeburg Rd., Orangeburg, NY 10962, USA; Melissa.Alldred@nki.rfmh.org; 3 Knoebel Institute for Healthy Aging, University of Denver, Denver, CO 80208, USA; 4 Department of Neurosurgery, CU Anschutz Medical Campus, 12700 E. 19th Ave., Aurora, CO 80045, USA

Down syndrome (DS), or trisomy 21, is the most common genetic cause of intellectual disability. Improvements in medical care, along with social enrichment, have led to a significant extension in lifespan and increased quality of life; however, they have also increased the prevalence of Alzheimer’s disease (AD). Many consider DS to be a form of accelerated aging, or that people with DS are highly prone to aging-related disorders, including vascular and neurological conditions. The current issue of the *Journal of Clinical Medicine* (JCM) contains a collection of papers depicting different aspects of aging with DS, from biological pathways involved in age-related conditions to the impact of lifestyle, or novel interventions that can reduce the effects of aging on the brain in people with DS. 

There is evidence from early onset AD (EOAD) in the general population that physical activity can slow the progression of AD pathology [1]. Therefore, Pape et al. (2021) [2] examined the impact of regular exercise on cognitive performance in adults with DS. They collected information and cognitive function measures in 214 adults with DS at baseline, and then at a 2-year follow-up. They found significant effects of moderate- or high-intensity exercise on clinical decline in this DS population and suggested that physical activity could have a long-term impact on the well-being of adults with DS, similar to findings in the general population. This is an important finding, and it is the first study to systematically measure the impact of exercise on aging in this vulnerable population.

The emergence of ultra-sensitive detection assays for blood biomarkers in recent years has revolutionized the biomarker field. This is especially important in populations with intellectual disabilities (ID), such as those with DS, for whom a lumbar puncture or MRI/PET imaging techniques may seem insurmountable. In addition, invasive research procedures on vulnerable populations are not permitted in certain countries or states, which could explain why only a few studies on CSF biomarkers exist for this population [3]. Montoliu-Gaya et al. (2021) [3] reviewed the current status of blood biomarkers, imaging, and CSF biomarkers for adults with DS, and concluded that sensitive new methods, such as the single molecule array (Simoa^®^, Quanterix), can provide a valuable source of biomarkers pertaining to the progression of dementia in adults with DS. The authors concluded that reliable and predictive biomarkers are especially important in adults with DS, due to the inherent variability in functionality that occurs earlier in life in those with ID, which poses unique challenges for the correct diagnosis of dementia. Several clinical cohorts in the US and Europe are working together to determine the best panels of blood biomarkers for adults with DS related to dementia. In Hendrix et al.’s (2021) [4] paper, the Longitudinal Investigation for Enhancing Down Syndrome Research (LIFE-DSR) Study reported early findings from a natural history study of adults with DS in the USA. The LIFE-DSR study consists of 11 sites, who are collectively recruiting 270 individuals with DS over the age of 25. The presented study contained data from the first 90 participants, demonstrated a strong, statistically significant effect of age on AD blood biomarkers, and suggested that conversion to AD pathology occurred at approximately 40 years of age. The clinical data included cognitive measures from the Severe Impairment Battery (SIB) and Down Syndrome Mental Status Examination (DS-MSE). This multi-site study offers important longitudinal information regarding conversion to AD and provides trial-ready participants for new interventions.

In the study by Dr. Raha-Chowdhury and collaborators (2021) [5], blood smears and *post-mortem* brain samples from individuals with AD and DS were analyzed using immunohistochemistry to explore the involvement of specific inflammatory proteins in hematopoiesis and cellular processes in DS-AD. They proposed that hepcidin, TREM2, and S100β play a critical role in immunoprotection, and are involved in communication between the periphery via macrophages and platelets and the brain parenchyma. These findings could lead to new targets for intervention in DS, as well as the general population with AD. Dr. Ledreux and collaborators (2021) [6] investigated small extracellular vesicles (exosomes) in blood from older adults with DS and AD (DS-AD). The investigators injected exosomes from DS-AD or controls into the brain of young adult wildtype (WT) mice, and found that the DS-AD exosomes, but not the control exosomes, gave rise to the accumulation of Tau-tangles in neurons, and some astrocytes in the mouse hippocampus. These studies may shed light onto how AD pathology spreads from region to region in the brain, and further the understanding of neuron-derived exosomes as valuable biomarkers for AD and other degenerative conditions.

The Perez/Mufson group (Moreno et al., 2021) [7] examined the neurochemical spatiotemporal development of hippocampal neurons in postnatal DS cases and compared these to neurotypical developing (NTD) cases using quantitative and qualitative immunohistochemistry. They reported that neuronal migration is compromised during hippocampal postnatal development in DS, independent of amyloid and tau pathology, and suggest that this likely contributes to intellectual disabilities observed in children with DS. Mhatre and collaborators (2021) [8] investigated the risk for AD in a population of males and females with DS, and, interestingly, discovered that males over the age of 60 were more than six times more likely to develop AD compared to age-matched females with DS. These findings were independent of ApoE status, ID level, and ethnicity, and raise interesting questions regarding the biological mechanisms involved in the striking finding regarding sex differences. Dr. Florence Lai et al. (2021) [9] performed a retrospective study on 339 adults with DS (125 who were cognitively stable (CS) and 214 with a diagnosis of AD). They examined the impact of autoimmune conditions, including alopecia, celiac disease, hypothyroidism, psoriasis, diabetes and vitamin B12 deficiency, as well as a systemic inflammatory condition, gout. They found that gout was associated with a significant delay in the age of AD onset by more than 2.5 years and concluded that systemic inflammatory conditions may play a role in the age of AD onset in DS. The Alldred/Ginsberg group (Alldred et al., 2021) [10] examined markers of Type II Diabetes in the Ts65Dn mouse model and the therapeutic benefit of maternal dietary choline during gestation and lactation. They found that adiponectin, but not leptin or insulin levels, were decreased in the frontal cortex of a mouse model of DS, and maternal choline treatment partially rescued adiponectin levels in the adult pups. The Barcelona group reported interesting new findings regarding sleep disturbances in the DS population (Giménez et al., 2021) [11]. They suggested that sleep disruption could influence the progression of dementia in individuals with DS and stressed the importance of investigating this clinical comorbidity more thoroughly. The Barcelona group also investigated the impact of epilepsy on older adults with DS (Altuna et al., 2021) [12] and concluded that late-onset epilepsy in DS is strongly associated with the development of symptomatic AD in this population. Their findings suggest that more than 50% of patients with DS and AD dementia may develop epilepsy, which has a specific clinical presentation in persons with DS, presenting as a form of generalized myoclonic epilepsy. The findings from the Barcelona research group and cohort of patients with DS and AD are important and have helped guide the clinical treatment of comorbidities of DS-AD for years. This is one of the best-characterized cohorts of adults with DS in the world. This cohort plays an important role in the European Clinical network for DS: Horizon 21. Finally, the research group from Cambridge University in the UK included an interesting review focused on the clinical and neuropathological features of sporadic and genetic forms of AD (Félez et al., 2021) [13], in which they compared the clinical and pathological aspects of genetic versus sporadic AD, focusing on DSAD and autosomal dominant Alzheimer’s disease (ADAD), compared with the more common late-onset form of the disease. They concluded that the variety of mutations causing ADAD could at least partially explain the wider range of phenotypes and progression of the disease. 

Overall, the goal of this collection of manuscripts is to present a comprehensive view of different and important aspects related to aging in people with Down syndrome. With this multidisciplinary approach and their synergistic expertise, the authors hope that advancements in the inclusion and treatment of age-related disorders in people with Down syndrome will become more common and achievable. The contributions to this Special Issue of *JCM* are a testament to the strong, inclusive, and widespread collaborative efforts in both the US and Europe regarding the improvement in clinical treatment of dementia in the DS population. 
